# Polish validation of the wisconsin stone quality of life questionnaire (POL-WISQoL)

**DOI:** 10.1007/s00345-024-05303-8

**Published:** 2024-10-23

**Authors:** Wojciech Tomczak, Wojciech Krajewski, Joanna Chorbińska, Łukasz Nowak, Katarzyna Grunwald, Adam Chełmoński, Jan Łaszkiewicz, Bartosz Małkiewicz, Tomasz Szydełko

**Affiliations:** 1 University Centre of Excellence in Urology, Wroclaw Medical University, Wroclaw, Poland; 2 Department of Minimally Invasive and Robotic Urology, University Centre of Excellence in Urology, Wroclaw Medical University, Wroclaw, Poland

**Keywords:** Urolithiasis, WISQOL, HRQOL, Kidney stone, Polish WISQOL

## Abstract

**Purpose:**

Urolithiasis significantly affects patient quality of life, yet the global standard of care predominantly focuses on achieving a stone free status, often ignoring patient reported outcomes. Currently, there are no specific measures available to assess the quality of life in the Polish population suffering from kidney stones. Therefore, this study aimed to develop and validate the Polish version of the Wisconsin Stone Quality of Life Questionnaire.

**Methods:**

The translation of WISQOL was carried out in accordance with the best available guidelines. Patients treated for kidney stones at a tertiary centre were recruited and completed both POL-WISQOL and SF36 questionnaires. Comprehensive analyses were conducted to assess internal consistency, inter-item and inter-domain correlations, as well as convergent and construct validity. Additionally, test-retest reliability was evaluated to ensure the accuracy and stability of the findings.

**Results:**

A total of 102 participants fully completed both questionnaires and were included in the analysis. The translated survey demonstrated excellent internal consistency (Cronbach’s coefficient 0.967) and significant convergent validity (Spearman’s correlation = 0.847, *p* < 0.001). Furthermore, an ANOVA with Tukey’s post hoc analysis revealed a significant decline in WISQOL scores between symptomatic and asymptomatic individuals, thereby confirming tool’s construct validity.

**Conclusion:**

POL-WISQoL turned out to be a valid disease specific health related quality of life measuring tool. Its widespread utilisation has the potential to shift the standard of care towards patient centered outcomes.

**Supplementary Information:**

The online version contains supplementary material available at 10.1007/s00345-024-05303-8.

## **Introduction**

Urolithiasis is a prevalent condition with its incidence being constantly on the rise for at least 30 years [1, 2]. Among others, this trend can be attributed to the increasing occurrence of risk factors, comorbidities that promote stone formation, and the use of drugs that alter urine composition [3–5]. Moreover, environmental factors along with global warming play their part [6]. Taken together, these factors further promote kidney stones recurrence, reaching up to 50% during 5yrs follow-up [7].

The clinical presentation of urolithiasis ranges from being entirely asymptomatic to causing severe physical and mental burden, particularly in recurrent stone formers. This has been displayed by utilising various questionnaires measuring health related quality of life (HRQoL) [8, 9]. Nevertheless, developing and implementing disease specific QoL questionnaires enables more precise symptom assessment and verification of their association with the disease, especially in highly comorbid patients. This approach facilitates a better understanding and improves the patient-oriented outcomes. Additionally, such a survey taken together with other clinical factors could allow for reduced number of procedures for recurrent stone formers as well as comprehensive treatment modalities comparison. The former is essential to preserve renal function which is frequently compromised by repeated interventions. Penniston et al. was the first to acknowledge and underline not only stone free rates but also patient reported outcomes by creating in 2013 Wisconsin Stone Quality of Life Questionnaire (WISQoL) [10]. Since publication, it has been widely utilised and proved to be a reliable tool, outperforming other unspecific HRQoL measures and being translated into many languages [10–15]. The absence of a Polish version of the WISQoL in current literature prompted this study, which aimed to develop and validate a Polish adaptation of the WISQoL.

## Materials and methods

### Translation and pilot testing

**Fig. 1:**
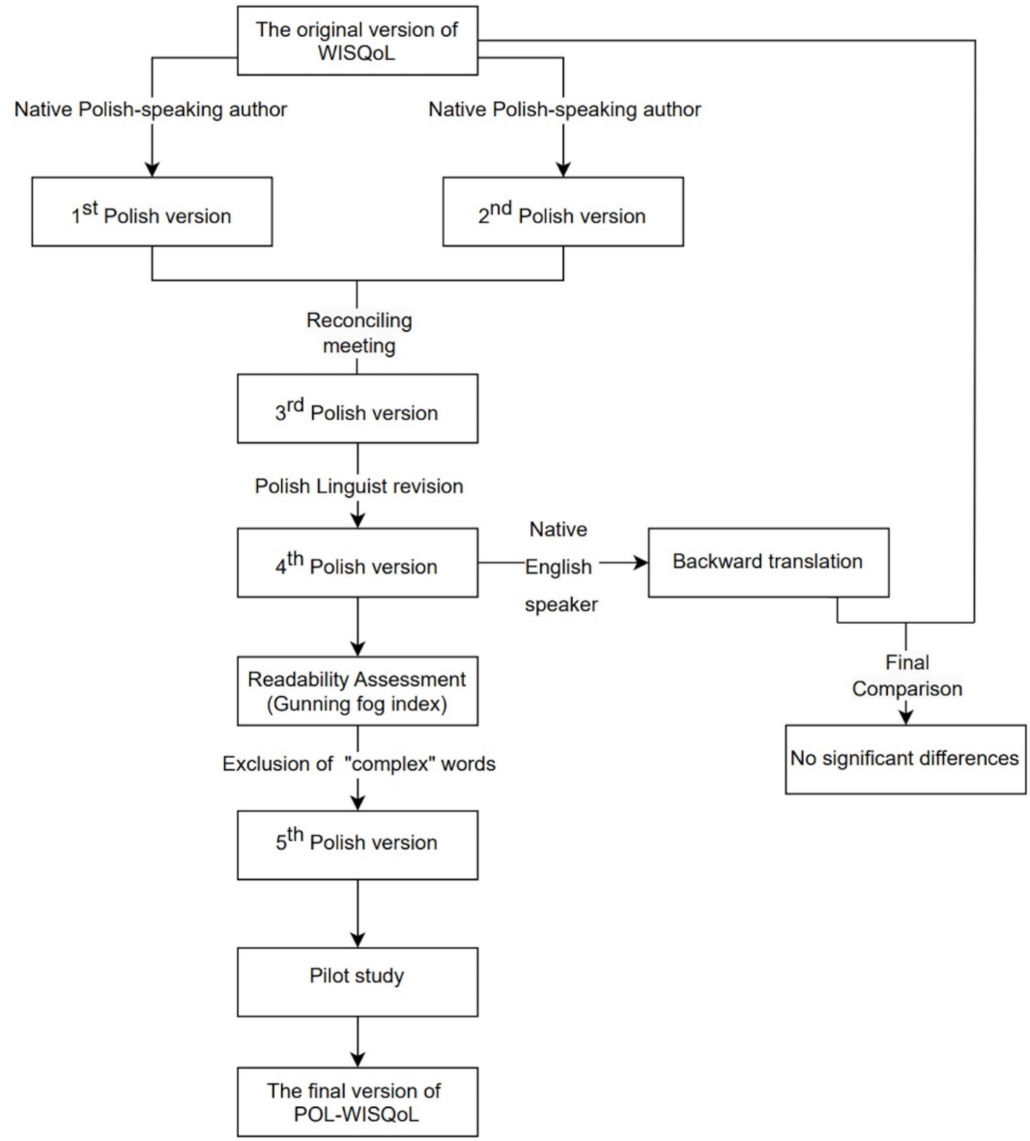
Step by step process for POL-WISQoL preparation

Translation and linguistic validation were implemented in accordance with the guidelines recommended by the original WISQoL creators [16]. Figure 1 illustrates in detail steps involved in the preparation of the POL-WISQoL questionnaire. Initially, two native Polish-speaking authors experienced in questionnaire validation conducted forward, concept-focused translations. Next, the reconciling meeting involving all authors, overseen by a Polish linguist was held. Derived version was subject to a backward translation performed by a native English speaker. The subsequent comparison of the original and backward translated questionnaires did not reveal any conceptual discrepancies. Before pilot testing POL-WISQoL was assessed with Gunning fog readability index. Words classified as “complex” were revised and paraphrased to enhance comprehension where possible. The pilot study included ten patients and was followed by a debriefing session with the revised version. Apart from small grammatical and punctuation changes no improvements have been made. The POL-WISQoL has been designed with the same format as the original questionnaire. The final version used for the validation of the POL-WISQOL is available at the WISQoL questionnaire creators.

### Used questionnaires

Short Form 36 (SF-36) is a commonly used questionnaire for assessing health-related quality of life in a variety of medical professions [17]. It is a generic measure consisting of 36 questions that evaluate eight distinct aspects of everyday functioning. These include Physical and Social Functioning, Role Limitations due to Physical Health and Emotional Problems, and levels of Energy. Additionally, the questionnaire quantifies Emotional Well-being, Pain, and General Health. All recorded answers are converted into 0-100 scale with total and domain scores being mean values of particular items.

WISQoL is the only disease-specific tool designed to evaluate the functional and symptomatic effects of urinary tract stones. It comprises 28 items that are organised into four functional domains: social (D1) and emotional (D2) impact, stone-related symptoms (D3), and vitality (D4). Each item is scored on a 5-point Likert scale, with a maximum score of 140 [10]. In both questionnaires reported QoL improves along with increasing scores.

### Study design and population

Patients admitted to the Urology Department of Wroclaw Medical University Hospital for endoscopic treatment of urolithiasis were prospectively recruited for the study. The following treatment methods were utilised: Retrograde Intrarenal Surgery (RIRS), Percutaneous Nephrolithotomy (PNL), Endoscopic Combined Intrarenal Surgery (ECIRS) and Simultaneous Bilateral Endoscopic Surgery (SBES). Only adults who were native Polish speakers and had at least a secondary level education were eligible to participate in this study. Individuals who were illiterate, mentally incapacitated, or diagnosed with conditions preventing informed consent completion were excluded from the study. In addition, a negative answer to 8.1. WISQoL item (“Did or do you currently have stones in your urinary system?”) at the admission to the hospital disqualified individuals from the study. Patients were enrolled in the study regardless of the symptoms presented.

After informed consent completion, patients completed SF-36 and POL-WISQoL questionnaires preoperatively. Of the 180 initially interested in participating in the study, only 157 signed informed consent (enrolment rate 87.2%). However, only 106 out of 157 people completed both questionnaires completely (completion rate 67.5%), with 4 of them marking NO in question 8.1. Successfully enrolled individuals were asked to complete the questionnaires again during a follow-up visit at the outpatient clinic 60 days postoperatively.

### Statistical analyses

Statistical analyses were conducted using Microsoft Excel version 16.86 and IBM SPSS Statistics software, version 27.0.1.0 A significance level was set at p value ≤ 0.05 for all calculations. Additionally, 95% confidence intervals (CIs) for Spearman’s rank correlations were computed using bootstrapping with 1000 iterations per each item tested.

Total and domain scores for POL-WISQoL and SF-36 were calculated in accordance with the scoring guidelines provided by creators of the questionnaires. Depending on type of distribution continuous variables were presented either as means with standard deviations (SD) or as medians with interquartile ranges (IQR). Categorical variables were presented as frequencies with percentages.

Reliability was assessed with internal consistency and test-retest analysis. The former was computed with Cronbach’s α for total and domain scores. Its coefficients were interpreted as unacceptable (< 0.50), poor (0.50–0.60), questionable (0.61–0.70), acceptable (0.71–0.80), good (0.81–0.90), and excellent (> 0.90). Test-retest reliability was evaluated by comparing total and domain scores from initial and 60-day postoperative visits in individuals who reported an unchanged health status. Spearman’s rank correlation was used. Unchanged health status was confirmed by identical answers to items 8.1 and 8.5 in both sets of responses.

The convergent validity of the POL-WISQoL and SF-36 total scores was assessed using Spearman rank correlation. Further evaluation included Spearman’s rho calculation for corresponding domains and items of both questionnaires. Item and inter-domain correlations for POL-WISQOL were also computed. The correlation coefficients were interpreted as follows: poor (≤ 0.20), fair (0.21–0.40), moderate (0.41–0.60), good (0.61–0.80), and excellent (> 0.80). Construct validity was estimated by mean scores comparison between patients who did and did not report currently having stone related symptoms (WISQoL item 8.2). This was established by one-way analysis of variance followed by Tukey’s honest significant difference tests for pairwise comparisons. Readability of the translated version was evaluated using the Gunning fog index.

## Results

A total of 102 participants fully completed the Polish versions of the WISQoL and SF-36 and were included in the analysis. All subjects were Polish, with a mean age of 52 (± 14.32). Among them, 58% were women. Table 1 complements the aforementioned data with the baseline clinical and demographic characteristics of the patients. The average preoperative POL-WISQoL score of our cohort was 89.56 ± 25.82. Detailed scores for each item are provided in Supplementary Table 1.

**Table 1:** Baseline sociodemographic patients characteristics

Total subjects number	102
Sex (%)	
Male	43 (42%)
Female	59 (58%)
Age (SD)	52.01 ± 14.32
BMI (SD)	27.62 ± 4.8
Mean stone diameter [mm]	14.71
Stone location %	
Renal pelvis	21
Upper calyx	6
Middle calyx	7
Lower calyx	28
Staghorn	11
Ureter	13
Multiple locations	16
Number of procedures	
No previous history	56
One	26
Two and more	20
Comorbidities Otherwise healthy	58
Hypertension	41
Diabetes mellitus	10
Dyslipidaemia	10
Hyperthyroidism	5
Depression	4
Other	8
Currently have kidney stones related symptoms	69 (67%)
Total POL-WISQoL score	89.56 ± 25.82
D1 (8–40)	26.47 ± 9.10
D2 (7–35)	22.14 ± 6.66
D3 (8–40)	24.21 ± 8.04
D4 (3–15)	8.66 ±3.37

The internal consistency of total and domain scores was evaluated by Cronbach’s α. All values were > 0.890. D3 and D4 achieved “good” internal consistency while D1, D2 and Total achieved “excellent” result. Test-retest reliability was calculated for subjects with unchanged health status with a correlation value ranging from 0.724 to 0.864. Detailed results of all included reliability measures are presented in Table 2.

**Table 2:** Internal consistency as Cronbach’s alpha and test-retest reliability for subjects with unchanged status (*n* = 56), all *p* values < 0.001

DOMAIN	Internal Consistency	Test-retest rho	95% CI
D1	0.935	0.864	(0.687–0.959)
D2	0.931	0.859	(0.717–0.948)
D3	0.890	0.724	(0.496–0.881)
D4	0.897	0.829	(0.641–0.934)
Total	0.967	0.858	(0.671–0.951)

**Fig. 2:**
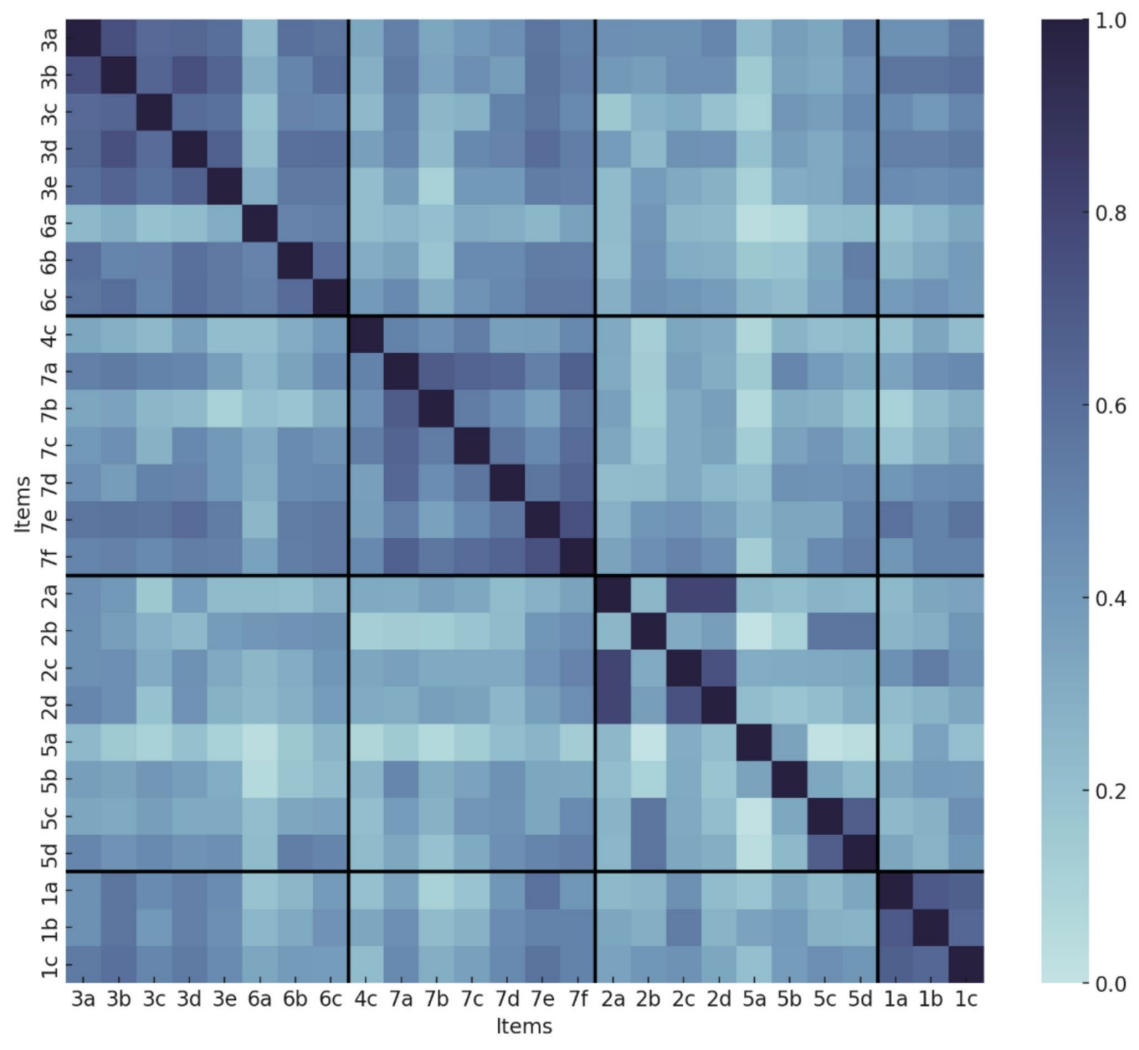
Item correlation graph, grouped as domains

The associations among all pairs of POL-WISQoL items are illustrated in Fig. 2. All items show positive correlation, with the lowest coefficient value of 0.248 and the highest being 0.853 (Fig. 2). Supplementary Table 2 contains all inter-item correlation values.

Inter-domain associations are presented in Table 3, showing “good” correlations between all pairs of domains (rho between 0.691 and 0.787). Additionally, all domains exhibited “excellent” correlations with the total score (rho ranging from 0.841 to 0.936).

**Table 3:** WISQoL Interdomain and domain - total correlations, all *p* values < 0.001, (95% CIs)

D1	0.936 (0.892–0.961)			
D2	0.898 (0.840–0.936)	0.787 (0.679–0.867)		
D3	0.903 (0.840–0.937)	0.786 (0.685–0.859)	0.755 (0.650–0.832)	
D4	0.841 (0.757–0.891)	0.786 (0.644–0.852)	0.691 (0.568–0.788)	0.715 (0.582–0.806)
	TOTAL	D1	D2	D3

**Fig. 3:**
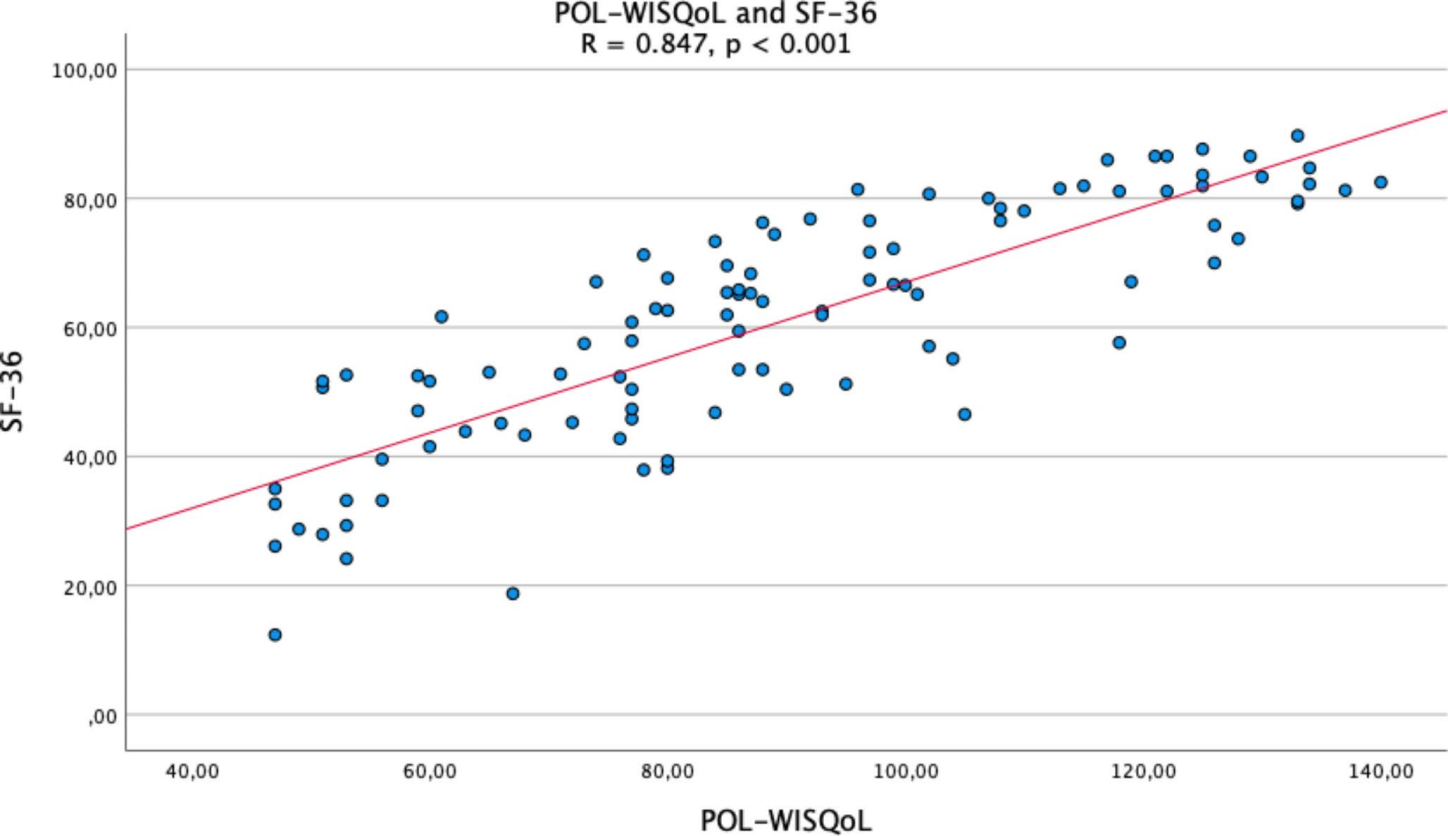
Preoperative correlation of POL-WISQoL and SF-36

The correlation of POL-WISQoL and SF-36 total scores was significant (rho = 0.847, CI95% 0.775–0.891, *p* < 0.001) (Fig. 3). Convergent validity was further verified by corresponding domains with Spearman’s rank correlation in accordance with the original validation study [18]. Additionally, corresponding items were evaluated as Gottstein et al. did (Table 4) [11]. Rho values for matching domains ranged from 0.589 to 0.724, indicating good concordance for pairs related to Pain and Physical symptoms. For those associated with Mental Health and Vitality, the values showed moderate agreement. Pairs of items associated with fatigue (1B-9G/I), concentration (3E-4D), social impact (3 A-6), and pain (5B-7), displayed significant correlation (range 0.503–0.643).

Construct validity was evaluated accordingly to the methods section. Mean total WISQoL scores differed between patients who did and did not report currently having stone related symptoms (item 8.2.) with p value < 0.002. The Gunning-Fog readability Index determined that comprehension of the POL-WISQoL requires 8.6 years of education.

**Table 4:** Convergent validity for corresponding items and domains, all *p* values < 0.001. *Reversed scoring

ITEMS	Spearman’s rho	CI95
WISQOL 1b - SF36 9 g	0.537	0.368–0.664
WISQOL 1b - SF36 9i	0.503	0.321–0.666
WISQOL 3e - SF36 4d	0.569	0.405–0.705
WISQOL 3a - SF36 6*	0.639	0.491–0.749
WISQOL 5b - SF36 7*	0.643	0.508–0.749
DOMAINS		
TOTAL - TOTAL	0.847	0.775–0.891
D1 - Role physical	0.643	0.508–0.754
D2 - Mental health	0.594	0.451–0.707
D3 - Body pain	0.724	0.607–0.815
D4 - Vitality	0.589	0.462–0.694

## Discussion

Urolithiasis is a complex condition with constantly increasing incidence and high recurrence rates. Both the disease and its treatment modalities can adversely affect HRQoL and compromise all areas of everyday functioning, particularly in recurrent stone formers [19]. Over the past decade, there has been a technological breakthrough in endourology. However, we believe that the era focused solely on stone-free outcomes has passed. It is time to provide comprehensive care for patients with urolithiasis, which includes assessing their expectations and quality of life.

This validation study was conducted in accordance with the established translation and validation protocols [16]. The results demonstrate that POL-WISQoL is a valid tool for assessing HRQoL in patients with urolithiasis. Furthermore, it is widely applicable to individuals regardless of their educational level. According to the Gunning-Fog readability index, translated questionnaire is comprehensible for individuals who have completed primary school.

Internal consistency was high across all domains indicating a strong correlation that aligns with findings from other validation studies [11–15]. Test-retest reliability scores were interestingly high in our population. However, this can be partially attributed to the fact that only patients with self-reported unchanged health status were included in the analysis. In our settings, these were either individuals waiting for another procedure or experiencing symptoms after achieving endoscopic stone free status.

The assessment of convergent validity involved calculating correlations between corresponding domains and items across both questionnaires. Our analysis showed significant positive correlations for all matched pairs (Table 4). It is worth noting that inter-item correlation can be performed using both versions of the SF36 questionnaire, as the matched items are identical. This presents a significant advantage for future validation studies in other languages, particularly only the first version of SF36 is available free of charge.

Since patients’ recruitment was performed upon admission to the hospital our cohort did not include stone-free individuals. The ones who stated otherwise (item 8.1) were excluded from the study. Therefore, our subgroup analysis included only symptomatic and asymptomatic comparison. Nevertheless, mean total scores between these two groups differed significantly proving its construct validity.

WISQoL scores typically improve within the first few months after the procedure, stabilising thereafter (e.g. 3–4 months in the study by Gottstein et al.) [11]. However, the timeframe for maximal HRQoL improvement varies in the literature. In our opinion, factors such as treatment modality and the chosen “exit strategy” are among the most influential. For instance, in complicated urolithiasis cases, a DJ stent is left in place to aid the passage of residual stone dust, facilitate mucosal healing, and prevent stricture formation. Nevertheless, the stent can cause irritative symptoms and pain, negatively impacting HRQoL. We contend that this may account for the delayed HRQoL improvement until its removal and the timeframe differences between centres.

This study comes with certain limitations. We are a tertiary referral centre often treating complicated cases from all over the country. Unfortunately, this is associated with the loss of follow-up, as patients who experience successful outcome (HRQoL improvement) tend to continue their follow-up at local outpatient clinics. In our cohort, the majority of patients attending follow-up appointments were symptomatic or scheduled for another procedure. Although this was a highly selected population it allowed us for test-retest reliability estimation.

Future investigations should focus on long term follow-up and HRQoL changes, ideally involving multiple urolithiasis treating centres to provide a broader perspective on a diverse patient populations and to track the tool’s performance over time.

## Conclusions

This study demonstrates that POL-WISQoL is a valid, reliable, and simple to comprehend tool. Its application will standardise research involving Polish population suffering from urolithiasis. This will help fill the gaps in data concerning the Central European population and further shift patient care towards outcomes that are oriented around the patient’s needs.

## Electronic supplementary material

Below is the link to the electronic supplementary material.

Supplementary Material 1

Supplementary Material 2: table 1. POL-WISQoL item scores.

Supplementary Material 3: table 2. POL-WISQoL inter-item correlation.

## Data Availability

No datasets were generated or analysed during the current study.
