# Supplementary figures and images for: Polish validation of the wisconsin stone quality of life questionnaire (POL-WISQoL)
Source: World J Urol. 2024 Oct 23;42(1):590. doi: 10.1007/s00345-024-05303-8 (PMC11499438; doi:10.1007/s00345-024-05303-8)

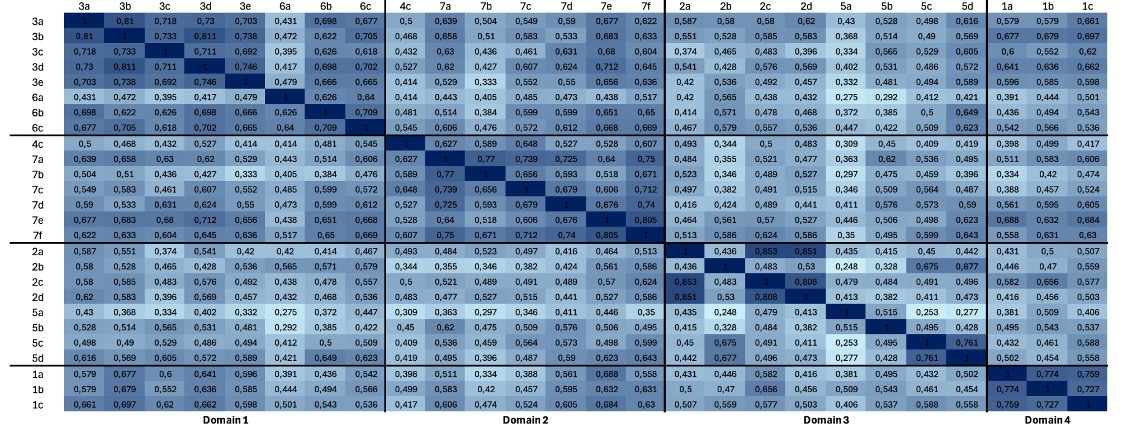

Supplement: Supplementary file 1 — Supplementary Material 1 [file 345_2024_5303_MOESM1_ESM.jpg]
