# Supplementary material for: Polish validation of the wisconsin stone quality of life questionnaire (POL-WISQoL)
Source: World J Urol. 2024 Oct 23;42(1):590. doi: 10.1007/s00345-024-05303-8 (PMC11499438; doi:10.1007/s00345-024-05303-8)
Supplement: Supplementary file 3 — Supplementary Material 3: table 2. POL-WISQoL inter-item correlation. [file 345_2024_5303_MOESM3_ESM.docx]

**SUPPLEMENTARY TABLE 1:**

| Item | Mean ± SD | median (range) |
| --- | --- | --- |
| 1a | 2.86 ± 1.23 | 3 (1-5) |
| 1b | 2.99 ± 1.08 | 3 (1-5) |
| 1c | 2.80 ± 1.36 | 3 (1-5) |
| 2a | 3.10 ± 1.58 | 3 (1-5) |
| 2b | 2.55 ± 1.46 | 2 (1-5) |
| 2c | 2.85 ± 1.47 | 3 (1-5) |
| 2d | 3.13 ± 1.48 | 3 (1-5) |
| 3a | 3.21 ± 1.45 | 3 (1-5) |
| 3b | 3.40 ± 1.32 | 3 (1-5) |
| 3c | 3.23 ± 1.26 | 3 (1-5) |
| 3d | 3.50 ± 1.32 | 4 (1-5) |
| 3e | 3.42 ± 1.31 | 4 (1-5) |
| 4a | 3.64 ± 1.11 | 4 (1-5) |
| 4b | 4.43 ± 0.85 | 5 (1-5) |
| 4c | 3.11 ± 1.10 | 3 (1-5) |
| 5a | 3.69 ± 1.09 | 4 (1-5) |
| 5b | 3.03 ± 1.08 | 3 (1-5) |
| 5c | 2.83 ± 1.14 | 3 (1-5) |
| 5d | 3.02 ± 1.27 | 3 (1-5) |
| 6a | 3.01 ± 1.54 | 3 (1-5) |
| 6b | 3.25 ± 1.39 | 3 (1-5) |
| 6c | 3.42 ± 1.34 | 4 (1-5) |
| 7a | 2.91 ± 1.24 | 3 (1-5) |
| 7b | 2.93 ± 1.13 | 3 (1-5) |
| 7c | 3.01 ± 1.14 | 3 (1-5) |
| 7d | 2.94 ± 1.09 | 3 (1-5) |
| 7e | 3.74 ± 1.12 | 4 (1-5) |
| 7f | 3.50 ± 1.11 | 4 (1-5) |

**Supplementary table 1**. POL-WISQoL item scores
